# Accumulation of heavy metals and antioxidant responses in *Pinus sylvestris* L. needles in polluted and non-polluted sites

**DOI:** 10.1007/s10646-016-1654-6

**Published:** 2016-03-31

**Authors:** Marta Kandziora-Ciupa, Ryszard Ciepał, Aleksandra Nadgórska-Socha, Gabriela Barczyk

**Affiliations:** Department of Ecology, University of Silesia, Bankowa 9, 40-007 Katowice, Poland

**Keywords:** Antioxidant response, Heavy metal, *Pinus sylvestris* L.

## Abstract

The purpose of this study was to determine the concentrations of heavy metals (cadmium, iron, manganese, lead and zinc) in current-year, 1-year old and 2-year old needles of *Pinus sylvestris* L. Trees were from three heavily polluted (immediate vicinity of zinc smelter, iron smelter and power plant) and three relatively clean sites (nature reserve, ecologically clean site and unprotected natural forest community) in southern Poland. Analysis also concerned the antioxidant response and contents of protein, proline, total glutathione, non-protein thiols and activity of guaiacol peroxidase (GPX) in the needles. Generally, in pine needles from the polluted sites, the concentrations of the metals were higher and increased with the age of needles, and in most cases, antioxidant responses also were elevated. The highest levels of Cd, Pb and Zn were found in 2-year old pine needles collected near the polluted zinc smelter (respectively: 6.15, 256.49, 393.5 mg kg^−1^), Fe in 2-year old pine needles in the vicinity of the iron smelter (206.82 mg kg^−1^) and Mn in 2-year old needles at the ecologically clean site (180.32 mg kg^−1^). Positive correlations were found between Fe, Mn and Pb and the content of proteins and NPTs, between Cd and non-protein –SH groups, and between Zn and proline levels. The activity of GPX increased under the influence of Mn, while glutathione levels tended to decrease as Mn levels rose. The data obtained show that the levels of protein and non-protein –SH groups may be useful in biological monitoring, and that these ecophysiological parameters seem to be good evidence of elevated oxidative stress caused by heavy metals.

## Introduction

Atmospheric pollution constitutes a major problem in urban environments (Al-Khlaifat and Al-Khashman 2007; Sawidis et al. 2011; Chen et al. 2016; Zhao et al. 2016). Pollutants containing trace metals are released from many different anthropogenic sources such as industry, and the combustion of fossil fuels in vehicles and energy plants (Sawidis et al. 2011). Heavy metal contamination can be exceptionally high in the vicinity of smelting operations and near mine tailings, i.e. materials left over after the process of separating the valuable fraction from the uneconomic fraction of an ore (Probst et al. 2009; Bothe 2011; Nadgórska-Socha et al. 2013b). The accumulation of those anthropogenic trace metals in plants has drawn considerable attention as a possible indicator of inorganic pollution of the environment, as plants respond directly to the state of the soil and air (Divan et al. 2009; Fowler et al. 2009; Serbula et al. 2013).

Environmental quality monitoring using biological material is commonly accepted as a reliable and affordable way of obtaining information on heavy metal contamination. The main advantage is the opportunity for long-term comparisons without the need for expensive equipment (Massa et al. 2010). The higher trophic plants most often used for biomonitoring in industrial and urban areas are coniferous and deciduous trees (Rademacher 2001; Piczak et al. 2003; Serbula et al. 2013). Their one great advantage is that they are long-lived, so that repeated investigations are possible over decades. They can thus be sampled systematically with standardized sampling and analytical techniques for comparative monitoring of the temporal distribution of trace elements. Trees are usually easier to identify than lower trophic plants and can be used as effective biomonitors to detect even low levels of anthropogenic pollutants. Although it can be difficult to distinguish between the amount of pollutants taken up from the soil to that deposited on the leaves, trees still reflect the cumulative effects of environmental pollution (Berlizov et al. 2007; Sawidis et al. 2011).

The Scots pine (*Pinus sylvestris* L.), the main forest-forming species in Europe, is sensitive to several industrial pollutants, including heavy metals (Micieta and Murín 1998; Rautio et al. 1998; Nieminen et al. 2004; Chudzińska et al. 2014). Pine needles, with their thick epicuticular wax layer, are most frequently used for biomonitoring of airborne pollution due to the possibility of both passive and active uptake by tissues from the atmosphere (Mingorance et al. 2007; Sun et al. 2009, 2010; Kuang et al. 2011; Serbula et al. 2013).

Heavy metal uptake and accumulation by plant tissues causes various morphological, physiological and biochemical responses (Doganlar and Atmaca 2011). Some metal ions are likely to remain in the cytoplasm and induce oxidative stress via the generation of reactive oxygen species (ROS), which hinder cell metabolism and lead to multiple toxic effects—such as lipid peroxidation, protein cleavage or DNA damage (Pongrac et al. 2009). Plants have evolved scavenging systems that control ROS using non-enzymatic antioxidants, such as glutathione, proline, ascorbic acid, carotenoids and non-protein compound rich in –SH groups, as well as enzymatic anti-oxidative systems. The levels of activity of anti-oxidative enzymes such as superoxidase dismutase, glutathione peroxidase, catalase and guaiacol peroxidase (GPX) have often been examined in research on heavy metal antioxidant defenses (Pongrac et al. 2009; Kafel et al. 2010; Boojar and Tavakkoli 2010; Nadgórska-Socha et al. 2013b). Antioxidant systems in plants may be used as early indicators of environmental stress on target organisms preceding morphological or ultrastructural damage, and such as warning indicators for the ecosystem (Białońska et al. 2007). Despite the many publications on the mechanisms of plant tolerance and the role of antioxidant systems in plant adaptation to high concentrations of heavy metals, woody plants (especially conifers) have not been studied sufficiently in this area (Ivanov et al. 2012).

The objectives of the present study were to establish the concentrations of heavy metals (Cd, Fe, Mn, Pb and Zn) in the needles of *P. sylvestris* L. growing naturally in polluted and non-polluted areas, as well as to determine and compare the levels of antioxidants (non-protein thiols, glutathione, proline), antioxidant enzyme (guaiacol peroxidase) and protein content as evidence of the measure of scavenging systems. In comparing the analytical results of heavy metals and ecophysiological changes in the pine needles, the following questions were investigated:

- Do the examined physiological parameters in the pine needles from the polluted sites differ from those at non-polluted sites?
- Are the examined physiological parameters good indicators of oxidative stress caused by heavy metals in plants living under field conditions?

## Materials and methods

### Study area

**Fig. 1:**
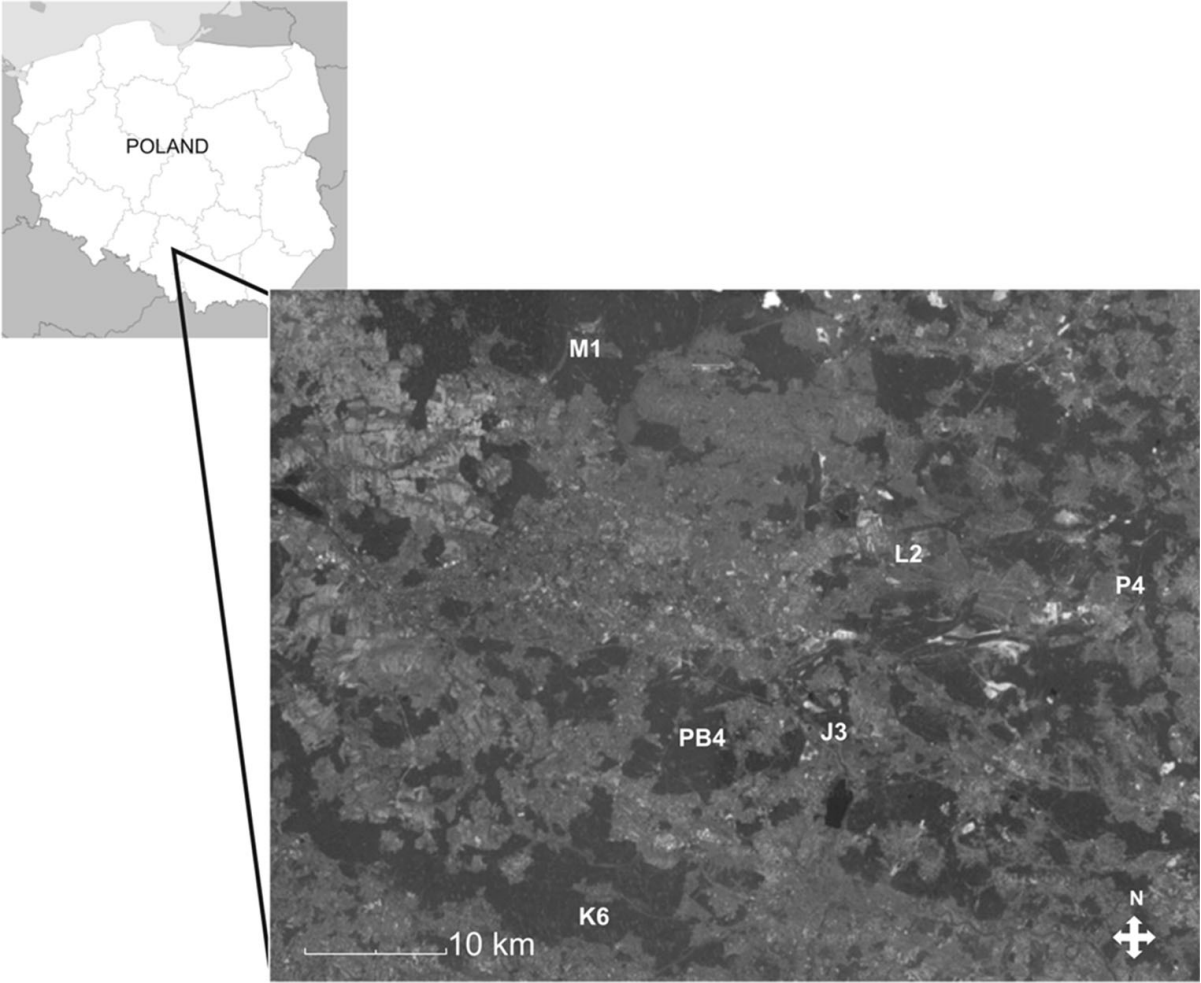
Location map of sampling sites (Kandziora-Ciupa et al. 2013)

The study was performed in typical pine forests located in three heavily polluted sites [immediate vicinity of a zinc smelter “Miasteczko Śląskie” (M), iron smelter “ArcelorMittal Poland S.A.” in Dąbrowa Górnicza-Łosień (L) and power plant “Jaworzno III” in Jaworzno (J)], and three potentially clean sites [(nature reserve “Pazurek” in Jaroszowiec Olkuski (P), ecologically clean site “Płone Bagno” in Katowice (PB) and an unprotected natural forest community in Kobiór (K)]. All the sites are situated in southern parts of Poland, in either the Śląskie or Małopolskie provinces (Fig. 1). Accurate characterization, data on soils and their contamination, and maps of the sites are given in a previous work by Kandziora-Ciupa et al. (2013).

### Sample collection

The determination of heavy metal content and biochemical analyses were conducted on the needles of *P. sylvestris* L. collected from 10 randomly chosen trees (35–40-years old) at each sampling site in mid-July of 2009. Taking into consideration individual variations in the trees, current-year (0), 1-year old (1) and 2-year old (2) needles (about 200 g for each age class) were randomly sampled from branches in the upper, middle and lower crown in the eastern, southern, western and northern directions of each tree, and pooled into each age class to form a composite sample, thus for all analyses there were three replicates per age class per site.

After collection, samples were placed in plastic bags, deposited on ice, transported directly to the laboratory and then frozen until analysis.

### Analysis of metal concentrations in leaf samples

In order to determine heavy metal concentrations in the pine needles, the plant material was washed with distilled water under a tap, and dried at 105 °C. After wet mineralization (as described in detail in Kandziora-Ciupa et al. 2013), the concentrations of Cd, Pb, Zn, Fe and Mn were established using inductively coupled plasma-atomic emission spectroscopy (Spectro Analytical Instruments). The quality of the analytical procedure was checked using a reference material (Certified reference Material CTA-OTL-1 Oriental Tobacco Leaves) with the same quantities of samples.

### Analysis of the biochemical parameters of the plants

Protein content was determined according to Bradford (1976) using bovine serum albumin as a standard.

Proline content was determined according to Bates et al. (1973), where 0.5 g of plant material was homogenized in 10 ml of sulphosalicylic acid (3 g per 100 ml), and the homogenate filtered through Whatman No. 2 filter paper. A reaction mixture containing 2 ml of homogenate, 2 ml ninhydrin acid and 2 ml of glacial acetic acid was incubated at 100 °C for 1 h. The reaction mixture was placed on ice and extracted with 4 ml of toluene. The absorbance was read at 520 nm using toluene as the blank. The proline content was expressed in μmol proline per g fresh weight.

To measure the total glutathione concentration (GSHt), 0.5 g of plant parts were homogenized in TCA (trichloroacetic acid, 5 g per 100 ml) together with 0.125 mM phosphate buffer (pH 6.3) with 6.3 mM EDTA, and centrifuged at 10,000*g* for 10 min at 4 °C. Supernatants were used for GSH determination using the DTNB-GSSG reductase recycling procedure according to Anderson (1985). The reaction mixture contained 0.2 ml of supernatant, 0.6 ml of 0.3 mM NADPH, 0.1 ml of 6 mM DTNB and 0.1 ml (0.5 IU ml^−1^) of glutathione reductase. The linear changes in the absorbance of the reaction mixtures were measured at 412 nm and the GSHt was expressed as μmol GSH g^−1^ fresh weight.

The content of non-protein thiols was estimated per the method described by Mass et al. (1987), where plant material was homogenized in a 5 vol g^−1^ mixture containing 5-sulphosalicylic acid (2 g per 100 ml), 1 mM EDTA and sodium ascorbate (0.15 g per 100 ml). The samples were centrifuged at 20,000*g* for 10 min at 4 °C. Following this, 0.5 ml of liquid supernatant, 0.5 ml of 1 M sodium phosphate buffer (pH 8.0) and 100 μl of 10 mM 5,5′-dithio-bis (2-nitrobenzoic acid) (DTNB) were put into the test tubes. The absorbance at 415 nm was read 1 min after the addition of DTNB. The number of non-protein SH groups was established based on a curve prepared using l-cysteine and was expressed in nmol –SH g^−1^ fresh weight.

For the analysis of guaiacol peroxidase fresh plant material was homogenized in a 100 mM phosphate buffer (pH 6.8). GPX activity was measured at 470 nm according to Fang and Kao (2000) and Liu et al. (2004) with guaiacol as the substrate. GPX activity was measured in a reaction mixture (3 ml) containing 50 mM phosphate buffer (pH 5.8), 1.6 μl H_2_O_2_, 1.5 μl guaiacol and 0.2 ml enzyme extract. Activity was calculated using the extinction coefficient (26 mM^−1^ cm^−1^) for tetraguaiacol and was expressed in μmol tetra-guaiacol g^−1^ fresh weight min^−1^.

### Statistical analysis

Data concerning the biochemical parameters and metal content were analysed (n = 3), checked for normality and equality of variance. The data was analyzed by ANOVA and the treatments were treated as independent variables. Significant statistical differences for all variables were established using Tukey tests, *p* < 0.05 (ANOVA; Statistica 10 package). We also calculated the Pearson correlation coefficient between the metal concentrations and biochemical parameters of the pine needles. Multiple regression equations were derived to determine which of the investigated metals influenced antioxidant levels and peroxidase activity in the needle samples from the study areas. The method of stepwise forward regression was applied. The equations concerned the the relationships between peroxidase (GPX) activity, the levels of glutathione (GSHt), non-protein thiols (NPTs), proline as well as protein, and the examined accumulated metals in the needles (Zn, Pb, Cd, Mn, Fe). Significance of differences was set at a level of *p* < 0.05.

## Results

### Heavy metal concentrations in *P. sylvestris* needles

The mean values of heavy metal concentrations found in the *P. sylvestris* needles were placed in the following descending order: Mn > Zn > Fe > Pb > Cd. There was a clear increase in the concentrations of the studied metals in the *P. sylvestris* needles from the polluted sites. The exception to this was Mn, where the highest levels were observed at the three non-polluted sites P, PB and K. Metal content in *P. sylvestris* needles also increased with increasing needle age class (Table 1). The highest Cd, Pb and Zn concentrations (respectively: 6.15, 256.49, 393.5 mg kg^−1^) were found in 2-year old pine needles collected at site M, the highest Fe content (206.82 mg kg^−1^) in 2-year old pine needles at site L, and Mn (180.32 mg kg^−1^) content highest in 2-year old pine needles from the non-polluted site PB (Table 1).

**Table 1:** The concentrations of heavy metals (mg kg^−1^ dw) in the needles of *P*. *sylvestris* (mean values ±SE, n = 3)

Site	Needles age class	Cd	Fe	Mn	Pb	Zn
M	M0	1.89 ± 0.01 c	49.39 ± 1.33 c	59.22 ± 0.20 b	31.23 ± 1.13 d	121.70 ± 5.03 c
	M1	4.39 ± 0.05 c	86.90 ± 1.02 d	67.07 ± 3.82 b	212.99 ± 5.50 c	290.50 ± 1.83 f
	M2	6.15 ± 0.24 b	143.25 ± 0.77 d	77.31 ± 3.05 a	256.49 ± 2.00 c	393.50 ± 1.50 f
L	L0	0.02 ± 0.00 a	51.29 ± 8.37 c	23.82 ± 4.57 a	5.98 ± 0.30 b	35.13 ± 3.30 b
	L1	0.17 ± 0.00 b	197.82 ± 0.67 f	39.77 ± 2.82 a	10.38 ± 0.94 ab	79.47 ± 0.03 e
	L2	0.17 ± 0.00 a	206.82 ± 2.33 e	62.07 ± 1.68 a	15.76 ± 0.30 b	142.87 ± 2.20 e
J	J0	0.03 ± 0.01 a	29.60 ± 1.38 ab	76.96 ± 3.87 c	7.64 ± 0.72 bc	38.30 ± 3.67 b
	J1	0.09 ± 0.02 a	72.24 ± 0.72 c	92.56 ± 1.87 c	14.83 ± 0.15 b	68.40 ± 1.00 d
	J2	0.15 ± 0.05 a	97.97 ± 12.05 b	127.21 ± 19.98 b	13.66 ± 1.51 b	113.37 ± 7.87 d
P	P0	0.10 ± 0.07 ab	59.29 ± 1.93 c	80.94 ± 7.62 c	8.70 ± 0.15 c	43.30 ± 0.13 b
	P1	0.15 ± 0.02 ab	62.00 ± 0.88 b	132.02 ± 9.07 d	13.00 ± 1.78 b	61.00 ± 1.87 c
	P2	0.22 ± 0.02 a	114.95 ± 0.73 c	173.47 ± 17.18 c	16.51 ± 0.15 b	75.35 ± 0.68 c
PB	PB0	0.07 ± 0.04 a	35.12 ± 3.17 b	84.86 ± 3.90 c	3.23 ± 0.02 a	21.00 ± 3.00 b
	PB1	0.08 ± 0.04 a	89.95 ± 0.43 e	159.51 ± 2.92 e	6.03 ± 0.35 a	55.05 ± 0.85 b
	PB2	0.18 ± 0.02 a	87.67 ± 1.68 b	180.32 ± 11.33 c	6.87 ± 1.68 a	61.43 ± 0.03 b
K	K0	0.18 ± 0.03 b	19.54 ± 0.65 a	58.57 ± 0.92 b	2.35 ± 0.62 a	12.11 ± 0.47 a
	K1	0.21 ± 0.01 b	47.42 ± 0.00 a	137.54 ± 3.82 e	4.25 ± 0.05 a	17.30 ± 0.20 a
	K2	0.37 ± 0.00 a	63.92 ± 0.00 a	167.56 ± 7.10 c	5.17 ± 0.02 a	19.18 ± 0.12 a
Sufficient or Normal^a^	–	0.05–0.2	–	30–300	5–10	27–150
Excessive or Toxic^a^	–	5–30	–	400–1000	30–300	100-400

The different letters denote significant differences between the particular metal concentrations in the same needles age class (*p* < 0.05)

^a^According to Kabata-Pendias and Pendias (2001)

### Biochemical status of the plants

**Fig. 2:**
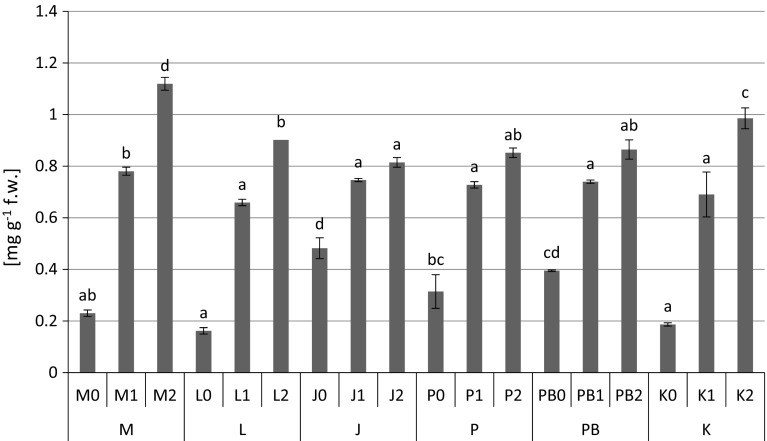
Protein contents (mg g^−1^ fresh weight) in *P. sylvestris* needles (mean values ±SE, n = 3). *Different letters above the columns* indicate significant differences in the same needles age class (*p* < 0.05)

**Fig. 3:**
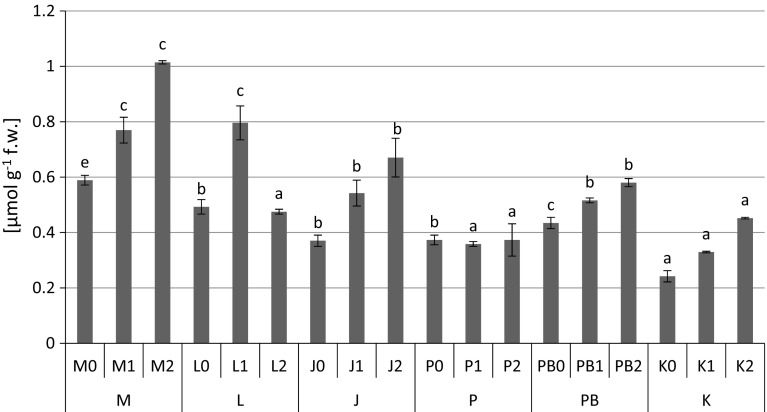
Proline contents (µmol g^−1^ fresh weight) in *P. sylvestris* needles (mean values ±SE, n = 3). *Different letters above the columns* indicate significant differences in the same needles age class (*p* < 0.05)

**Fig. 4:**
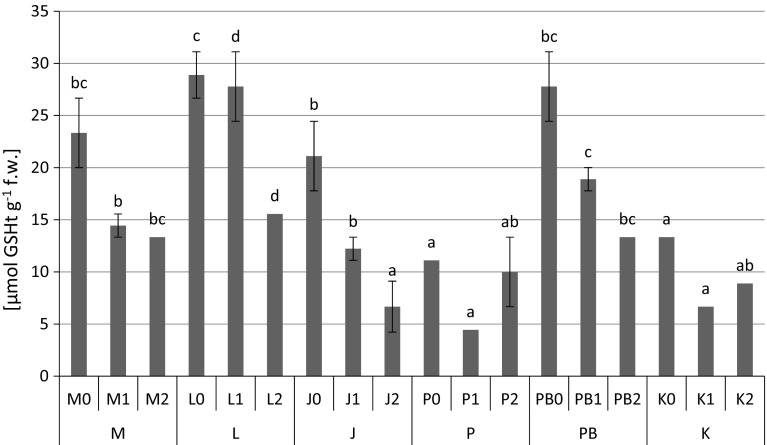
Total glutathione contents (µmol GSHt g^−1^ fresh weight) in *P. sylvestris* needles (mean values ±SE, n = 3). *Different letters above the columns* indicate significant differences in the same needles age class (*p* < 0.05)

**Fig. 5:**
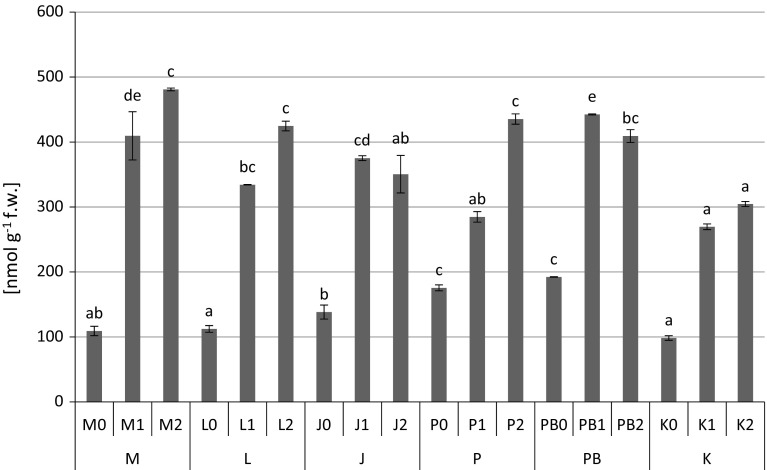
Non-protein –SH groups content (nmol –SH g^−1^ fresh weight) in *P. sylvestris* needles (mean values ±SE, n = 3). *Different letters above the columns* indicate significant differences in the same needles age class (*p* < 0.05)

**Fig. 6:**
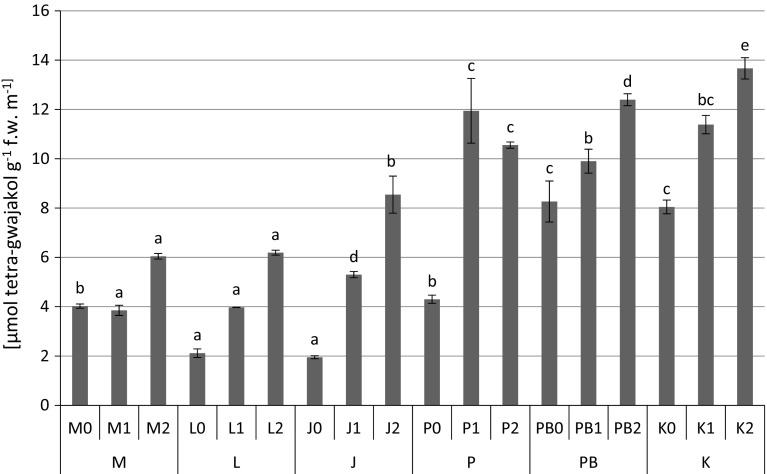
Guaiacol peroxidase activity (µmol tetra-guaiacol g^−1^ fresh weight min^−1^) in *P. sylvestris* needles (mean values ±SE, n = 3). *Different letters above the columns* indicate significant differences in the same needles age class (*p* < 0.05)

The highest content of proteins (1.12 mg g^−1^ fresh weight) (Fig. 2), proline (Fig. 3) and non-protein –SH (481 nmol –SH g^−1^ fresh weight) (Fig. 5) groups were found in *P. sylvestris* needles collected from the contaminated areas (M) in comparison to the potentially clean sites. The highest accumulation of GSHt was found in *P. sylvestris* needles from the site L—28.89 µmol GSHt g^−1^ fresh weight (Fig. 4). There were no clear differences in glutathione content between the polluted and non-polluted sites (Fig. 4). Guaiacol peroxidase activity was by far highest in the pine needles from the non-polluted site K (8.04–13.66 µmol tetra-guaiacol g^−1^ fresh weight min^−1^), where there were also high Mn concentrations (Fig. 6; Table 1).

The high multiple corrected correlation coefficients for the values presented in Table 2 suggest that the models (except GSHt) describe the majority of the variations for each dependent variable (antioxidants and GPX). Fe, Mn and Pb had a positive influence on protein content and NPTs (additionally, we found a positive correlation between non-protein –SH groups and Cd content in pine needles). Moreover, a positive correlation occurred between Mn contents and GPX as well as Zn concentration and proline content in the needles (Table 2). Only Mn had a negative effect on GSHt. These results are supported by the positive Pearson’s correlation coefficient values obtained between the examined antioxidants and GPX and the above mentioned metals (Table 3).

**Table 2:** Multiple regression equations (*p* < 0.05)

	R^2^
Protein = −0.061 + 0.539 (Fe) + 0.691 (Mn) + 0.416 (Pb)	0.863
Proline = 0.38 + 0.817 (Zn)	0.646
GSHt = 25.06 − 0.62 (Mn)	0.339
NPTs = −10.899 + 0.96(Cd) + 0.598(Fe) + 0.597(Mn) + 1.35(Pb)	0.929
GPX = 0.80 + 0.866(Mn)	0.750

**Table 3:** The correlation coefficients between metal concentration and antioxidant measurements in the in the needles of *P*. *sylvestris* (*p* < 0.05)

	Cd	Fe	Mn	Pb	Zn
Protein	0.34	0.57	0.54	0.40	0.47
Proline	0.73	0.55	NS	0.73	0.81
GSHt	NS	NS	−0.59	NS	NS
NPTs	0.34	0.67	0.45	0.42	0.52
GPX	NS	NS	0.85	NS	−0.27

*NS* not significant

In most cases (except GSHt concentration) there was an age-related increase in content or activity of investigated biochemical parameters. The total glutathione pool was mostly lower in the older pine needles.

## Discussion

The accumulation of heavy metals in forest vegetation is highly variable and affected by both physiological and environmental factors. Bioaccumulation of heavy metals by plant foliage presents a health risk to wildlife and potentially to humans (Pacyna and Pacyna 2001; Kabata-Pendias and Pendias (2001); McGee et al. 2007). Higher plants are believed to be less tolerant to increased concentrations of heavy metals, and to accumulate these elements and survive in highly heavy metal contaminated soils. The most toxic metals for higher plants in high concentrations in tissues include Pb and Cd (Ots and Mandre 2012).

Significantly higher levels of Cd, Pb and Zn were found in the pine needles collected at the site located near a zinc smelter (M), in comparison with all the other sites (Table 1). The levels of these three metals exceeded values considered as normal (according to Kabata-Pendias and Pendias 2001) and rose with the increasing age class of the pine needles. A similar pattern was observed by examining the contents of heavy metals in the leaves of *Vaccinium myrtillus* L. growing at the same sites (Kandziora-Ciupa et al. 2013). Also Chudzińska et al. (2014) examined 1-year-old *P. sylvestris* needles from the vicinity of the same zinc smelter in Miasteczko Śląskie and determined similar amounts of Cd, Pb and Zn to our investigations. At the non-polluted sites, the concentrations of Cd, Pb, Zn, Fe and Mn were similar to other non-polluted sites reported in literature (Parzych and Sobisz 2012; Serbula et al. 2013).

One of the mechanisms in plants affected by heavy metals is protein synthesis. It is known that soluble protein content is an important indicator of the physiological status of the plant (Doganlar and Atmaca 2011). In this study we noticed an increase in protein content in pine needles from polluted sites, especially in 1-year old and 2-year old needles (Fig. 2), and a positive correlation between Fe, Mn and Pb (Tab. 2). In a previous work, we found a strong positive correlation between protein content and Cd, Pb and Zn concentrations in the leaves of *V. myrtillus* (Kandziora-Ciupa et al. 2013). Also Doganlar and Atmaca (2011) reported an increase in total soluble protein content related to Al concentration in the leaves of *P. orientalis*, and Zn concentration in the leaves of *M. azaderach*. Mesmar and Jaber (1991) found an increase in total protein content related to an increase in lead concentration in wheat and lentil, and Singh et al. (2006) reported increased protein content in *Oryza sativa* under Cd stress. This increase may be a specific mechanism by which cells compensate for the protein content that has been deactivated due to metal binding, and also the effect of the increasing content of stress proteins (Mesmar and Jaber 1991; Seregin and Ivanov 2001).

Enzymatic and non-enzymatic antioxidants are important in plant defenses against heavy metals (Gill and Tuteja 2010). Moreover a field study concerning activity of oxidative enzymes may be helpful in evaluating these as biomarkers in the future, and may also help in understanding the defense mechanism of plants under chronic heavy metal stress (Nadgórska-Socha et al. 2013b).

Low molecular compounds, such as proline, play an important role in cell protection against ROS-induced damage (Kartashov et al. 2008; Ivanov et al. 2013). Metal-induced proline accumulation has been observed, and it has been suggested that this amino acid acts as a radical scavenger or it is involved in metal ion chelation (Andrade et al. 2009). In plants, proline constitutes <5 % of the total pool of free amino acids under normal conditions and 80 % of the total amino acid pool under stress (Kumar et al. 2010). An increase in proline levels in response to the impact of heavy metals is a widespread phenomenon among many plant species and has been noted by many investigators (Schat et al. 1997, Chen et al. 2004; Tripathi and Gaur 2004; Balestrasse et al. 2005; Sharma and Dietz 2006; Sun et al. 2007; Abdel-Latif 2008).

Our tests found a significant effect of Zn on the accumulation of proline in the needles of *P. sylvestris* (Table 2). The highest proline content was observed in pine needles in the vicinity of the zinc smelter (site M) and the iron smelter (site L) (Fig. 3). An increase in free proline level during environmental contamination was also found in *Philadelphus coronarius* leaves (Kafel et al. 2010). Ivanov et al. (2012) demonstrated a significant proline accumulation in the roots, hypocotyls and needles of Scots pine seedlings in response to the action of 150 µM Pb^2+^, and in the needles, cotyleodons and stems at 150 µM Zn. Nikolić et al. (2008) found a significantly higher accumulation of free proline in the roots than the leaves of hybrid poplar under the influence of Cd. Zengin and Munzuroglu (2005) registered a significant increase of proline content in bean leaves treated with Pb, Cu, Cd and Hg. Sharma and Dietz (2006) reported an increase in free proline accumulation in response to the increased accumulation of heavy metals in several examples of flowering plants.

Glutathione is a very important low molecular weight biological thiol crucial for the detoxification of heavy metals; which also acts as a precursor for the synthesis of phytochelatins (Yadav 2010). GSH creates complexes with heavy metals, and an induction of glutathione has been documented in plants as a response to heavy metal stress. The changes in GSHt level are dependent on the metal and the part of the plant (Arya et al. 2008; Nadgórska-Socha et al. 2013a).

As in previous research (Kandziora-Ciupa et al. 2013) on bilberry leaves, we found a decline in GSHt due to increased concentrations of Mn (Table 2). We observed a decrease in glutathione with increasing age class of the pine needles, also we found a negative correlation coefficient between the content of Mn and GSHt which was confirmed by the regression equation (Fig. 4; Tables 2, 3). In many cases, exposure to heavy metals results in reduced glutathione levels (Ni and Zn in *Cajanus cajan*—Madhava Rao and Sresty 2000; Cd in pine—Schützendübel et al. 2001; Pb in *Vicia faba* and *Phaseolus vulgaris*—Piechalak et al. 2002; Cd in *Triticum aestivum* L.—Lin et al. 2007; Cd in young *Picea omorika* (Pančić) Purk.—Dučić et al. 2008; Pb in *Raphanus sativus*—El-Beltagi and Mohamed 2010). This decline in the glutathione content in plants may result from inhibition of the enzymes involved in glutathione synthesis by toxic metal ions. In addition, the reduction in glutathione pool may also be considered to play some role in the synthesis of phytochelatins (PCs) (Madhava Rao and Sresty 2000). Sudhakar et al. (2006) showed that exposure of *Hydrilla verticillata* (L.f.) Royle to high doses of copper led to a decrease in GSH as a result of increased phytochelatin synthesis. Boojar and Tavakkoli (2010) found a similar dependency in *Alhagi camemelorum*. Fisch and de Vos et al. (1992) also indicated a decrease in GSHt caused by Cu in *Silene cucubalus* and an elevation in PC levels.

In plant cells, low-molecular weight compounds other than glutathione containing –SH groups can exist, such as phytochelatins, metallothioneins, thionins and defensins (Nadgórska-Socha et al. 2013b). Molecules containing sulphur, which exist in a wide variety of cells, may fulfill different functions and may be independently regulated (Mishra et al. 2009). Non-protein compounds rich in –SH groups are involved in metal detoxification and/or metal allocation between different organs of the plant, because their main task is binding of metal ions and forming non-toxic complexes with metals which are transported from the cytoplasm into the vacuole (Andrade et al. 2010; Yadav 2010).

In our study, an increase in NPT content was noticed in the needles of *P. sylvestris* grown at the most polluted sites (M and L), especially in the 2-year old needles, and also at clean sites where we noted high concentrations of Mn (Fig. 5). Additionally, the content of non-protein thiols was positively related with concentrations of Cd, Fe, Mn and Pb (Table 2). Our results were similar to Nadgórska-Socha et al. (2011) who observed an increase in non-protein –SH group content in the leaves of *Silene vulgaris* populations on a substrate with Cd and a combination of metals (Zn, Cd and Pb). Also Mishra et al. (2009) and Pongrac et al. (2009) found a strong positive correlation between NPT and Cd content in *Ceratophyllum demersum* L. and *Thlapsi praeox*. The increase in non-protein thiol levels indicates an ability to tolerate the cellular metal load (Mishra et al. 2006).

Peroxidases are antioxidant enzymes which are significant in plant growth and development. Activities of these enzymes are changed under both biotic and abiotic stress conditions, and so are used as a potential indicator of metal toxicity (Radotić et al. 2000; Macfarlane and Burchett 2001; Baycu et al. 2006; Doganlar and Atmaca 2011; Kandziora-Ciupa et al. 2013). For example, in Radotić et al. (2000), peroxidases were used as a potential biomarker for sublethal toxicity in spruce seedlings.

In this study on pine needles, the highest GPX activity was observed at the clean sites, where we also detected the highest content of Mn. The lowest values of guaiacol peroxidase activity were observed at the polluted sites M and L (Fig. 6). An increase in GPX activity in *P. sylvestris* needles was observed under the influence of higher levels of Mn (Table 2).

Many authors have reported increasing GPX activity with increasing heavy metal content (Macfarlane and Burchett 2001; Markkola et al. 2002, Hagemeyer 2004; Nadgórska-Socha et al. 2008; Kafel et al. 2010; El-Beltagi and Mohamed 2010; Doganlar and Atmaca 2011; Nadgórska-Socha et al. 2013a, b). However, similar to our work, Baycu et al. (2006), in their examination of peroxidase activity in the leaves of deciduous trees growing in urban parks in Turkey, also observed both increased and decreased peroxidase activity. Sometimes chronic metal-induced stress did not cause a measurable increase in oxidative stress (Słomka et al. 2008). Results obtained by Pongrac et al. (2009) reported no change in GPX activity in *Thalspsi praecox* and *T. caerulescens* in the presence of Cd and Zn; similar results were observed by Gratão et al. (2008) in the leaves, roots and fruits of the tomato grown in conditions of cadmium contamination. Słomka et al. (2008) demonstrated comparable or lower activity of peroxidase in the leaves of *Viola tricolor* in metalliferous versus clean areas. Sandalio et al. (2001) noticed a decrease in peroxidase activity in pea plants under the influence of Cd. Stress intensity may be linked to an increase or decrease in oxidative metabolism. The modulation of antioxidant levels constitutes an important adaptive response in withstanding adverse conditions, and maintenance of a high antioxidant capacity in cells is linked to increased tolerance against stress, for instance to heavy metal contamination (Thomas et al. 1999, Baycu et al. 2006). For this reason, varying antioxidative enzyme activity in response to heavy metals has often been found (Baycu et al. 2006).

It is difficult to draw clear major conclusions from research carried out in the wild, and we do realize that the examined ecophysiological parameters are influenced by many factors, and that it is not possible to take them all into account during in situ studies. Nevertheless a multi-parametic approach provides an understanding of the diverse responses and effects of exposure to contaminants, and the effective risks posed for different plant species (Stobrawa and Lorenc-Plucińska 2007; Fonseca et al. 2011; Oliva et al. 2012; Kandziora-Ciupa et al. 2013).

Based on our study we can conclude that: an increase in content of protein and non-protein –SH groups in the needles of *P. sylvestris* from polluted sites is most likely evidence of enhanced oxidative stress caused by heavy metals. Therefore, these ecophysiological parameters seem to be good biochemical markers to predict heavy metal pollution.

## Abbreviations

NPTs: Non-protein thiols
GSHt: Glutathione total
GPX: Guaiacol peroxidase
